# Impact of activity outcome and measurement instrument on estimates of youth compliance with physical activity guidelines: a cross-sectional study

**DOI:** 10.1186/s12889-016-2901-8

**Published:** 2016-03-03

**Authors:** Paul R. Hibbing, Youngwon Kim, Pedro F. Saint-Maurice, Gregory J. Welk

**Affiliations:** Iowa State University, 237 Forker Building, Ames, IA 50011 USA; MRC Epidemiology Unit, University of Cambridge School of Clinical Medicine, Cambridge, UK; University of Minho, Braga, Portugal

**Keywords:** Guideline, Physical activity, Youth, Monitor

## Abstract

**Background:**

The national physical activity guidelines (PAG) in many countries recommend that youth accumulate 60 min or more of moderate-to-vigorous physical activity (MVPA) daily (PAG-MVPA). A daily target of ≥ 11,500 steps/day has been proposed as a step count alternative to this guideline (PAG-Steps). Contemporary activity monitors are capable of estimating both MVPA and steps, but it is not clear how these units compare when used to evaluate compliance with the national PAG. The purpose of this study was to compare prevalence estimates of meeting the PAG-MVPA and PAG-Steps using two commonly used monitors, the ActiGraph (AG) and SenseWear Armband (SWA).

**Methods:**

A sample of 69 children (25 girls and 44 boys) aged 9–16 years each wore a wrist-mounted AG and a SWA over a one-week period. Days with ≥10 h of wear time for both monitors were included in the analysis. Estimates of time spent in MVPA were obtained using the Crouter equation for the AG and from proprietary algorithms for the SWA. Step counts for the AG and SWA were directly obtained from the respective software. The prevalence of meeting the PAG-MVPA and PAG-Steps was compared within each monitor, using Cohen’s kappa (κ) statistic. Agreement was similarly assessed between monitors using each guideline individually.

**Results:**

When assessed with the AG, the prevalence of meeting PAG was substantially higher for the PAG-MVPA (87.2 %) than for the PAG-Steps (54.2 %), with fair classification agreement (κ = 0.30) between the two guidelines. Higher prevalence rates were also observed for the PAG-MVPA (83.6 %) than for the PAG-Steps (33.8 %) when assessed using the SWA, but the prevalence rates and classification agreement (κ = 0.18) were lower than the values from the AG. Classification agreement between AG and SWA was lower for the PAG-MVPA (κ = 0.42) than for the PAG-Steps (κ = 0.55).

**Conclusions:**

The results show differential patterns of compliance with the PAG-MVPA and PAG-Steps, as assessed by the AG and SWA. Additional research is needed to directly evaluate and compare findings from public health research based on different guidelines and measurement methods.

## Background

Regular participation in physical activity (PA) has been shown to reduce risk factors for developing cardiovascular disease, type II diabetes, and a number of other chronic conditions in children and adolescents [1]. This has prompted the United States Department of Health and Human Services and other international institutions to establish physical activity guidelines (PAG) – with most recommending at least 60 min of moderate-to-vigorous physical activity (MVPA) as a daily target for children and adolescents (PAG-MVPA) [2–5]. The availability of accurate and easy-to-use methods for assessing time spent in MVPA is critical for evaluating compliance with the PAG-MVPA, which is a common outcome in many public health research applications. Self-report instruments have documented limitations in this respect, which has spurred the development of new tools and the release of a number of electronic monitoring devices [6].

Accelerometer-based PA monitors have become a staple of PA research because of their potential to characterize activity levels based on body segment accelerations. The ActiGraph (AG) (ActiGraph, Pensacola, FL, USA) and the SenseWear Armband (SWA) (BodyMedia, Inc., Pittsburgh, PA, now merged with Jawbone®, San Francisco, CA) are two commonly used tools in public health research [7–10]. There are calibrated algorithms in place for these monitors to estimate time spent in MVPA [11] as well as other activity/inactivity-related outcomes. Numerous studies have used these methods to evaluate compliance with the PAG-MVPA. Pedometers are also commonly used in research with youth and have been used in the development of several steps/day thresholds designed to approximate compliance with the PAG-MVPA [12, 13]. The advent of the accelerometer has contributed to this line of research [14], as most accelerometer-based PA monitors estimate both MVPA time and step counts. Most recently, AG data from the National Health and Nutrition Examination Survey (NHANES) were used to derive 11,500 steps/day as a generally applicable target for accelerometers (PAG-Steps) [15].

In spite of the potential benefits attending these technological advances, the use of different PA recommendations (i.e. PAG-MVPA versus PAG-Steps) and/or different activity monitors (i.e. AG versus SWA) can potentially lead to different estimates of youth activity levels, making it difficult to establish a solid scientific base for public health research on youth PA. Understanding this interplay of factors requires multi-faceted investigations. Therefore, the purpose of this study was to examine the impact of different guidelines and different monitors on evaluations of PA behavior. This was accomplished by comparing adherence to the PAG-MVPA and the PAG-Steps between concurrently worn AG and SWA monitors.

## Methods

### Study design

A sample of children and adolescents (9–16 years of age) wore both an AG GT3X+ on the non-dominant wrist and a SWA Mini on the back of the non-dominant upper arm for 7 days, removing them for swimming, bathing, or other submersion-based water activities. All parents of participants gave written informed consent before the study began, and all participants provided written assent. Participants were recruited either from an existing database of previous studies done in our lab, or by word-of-mouth. Participants were required to complete two visits. At the initial visit, demographic information was recorded (birthdate, handedness, and height and weight measured by a portable stadiometer and scale, respectively), and the monitors were distributed. Verbal instruction was given regarding how to wear the monitors, and pictures illustrating proper placement were also included in the youth assent form. The monitors were retrieved one week later, at the second visit. All study procedures were approved by the Institutional Review Board of Iowa State University.

### Instruments

The AG GT3X+ is an objective PA monitor equipped with two sensors. The first detects ambient light, and the second is a micro-electro-mechanical-system accelerometer with a dynamic range of ±6 g. The monitor can be initialized to record data at sampling rates from 30 to 100 Hz, in 10-Hz increments. It is lightweight, and is typically worn at the hip or on the wrist. When downloaded, a default filter eliminates signal outside the range of normal human movements, yielding a unit of measurement known as activity counts. The corresponding software includes algorithms to summarize tri-axial data, including a single figure known as vector magnitude, and can also derive inclinometer information and step counts from the recorded accelerations.

The SWA Mini is a multi-sensor PA monitor that samples skin temperature, galvanic skin response, and heat flux in addition to triaxial acceleration. It is small, minimally invasive, and currently suitable for placement on the posterior aspect of either upper arm. Proprietary algorithms in the corresponding software estimate minute-by-minute energy expenditure and step counts using the monitor data and the wearer’s demographic information (birthdate, height, weight, BMI, sex, handedness, and smoking status).

Step detection is governed by proprietary algorithms for both the AG and SWA. Limited information is available regarding the algorithm for the AG [16], but the user’s manual states that only vertical axis accelerations are used, and are first cleaned in order to filter out baseline noise. No information is available about the specific step counting algorithms for the SWA but other documentation emphasizes the use of pattern recognition methods for classifying activities. Both instruments have been commonly used in child populations [9, 10, 17–19], making them excellent representative choices to evaluate differences in PAG compliance between estimates of MVPA and steps.

It is important to point out that our choice to use wrist-worn triaxial AG monitors contrasted with the uniaxial hip-worn AG monitors used to develop the PAG-Steps [15]. The decision to use the AG was primarily related to its widespread use in the field [7]. While some applications and methods still utilize the hip-worn position, there has been a strong movement towards wrist-worn monitors – including within national surveillance studies such as the NHANES. Several studies have examined the impact of this transition to wrist-worn monitors for both MVPA [20] and steps [21] as well as estimates of sedentary behavior [22]. We used the non-dominant wrist in the present study in order to avoid potential movement artefacts from common dominant-biased activities such as writing or eating [20]. However, it should be noted that artefactual movement is a risk at both wrists, and it is unclear whether results differ by attachment to the dominant versus non-dominant wrist [10].

Taken together, using the increasingly more common placement (i.e. the wrist) over the more established hip placement [20, 21] allowed us to directly compare monitor selection and outcome measure in a more relevant way. Understanding the impacts of these options is important to advance public health surveillance of PA.

### Data processing

PA estimates for the AG were derived from the cut-points developed by Crouter et al. [10]. The AG monitors were initialized at 100 Hz and processed in 5-s epochs with the normal filter applied. SWA data were processed using the SWA’s software version 8.0 (coupled with proprietary algorithms version 5.2). After processing, AG estimates were collapsed (i.e. summed) into 60-s summaries (maintaining 5-s resolution) to facilitate temporal matching with the data from the SWA. This process should not be confused with reintegration, in which the data are reformatted, and the estimates are re-calculated, resulting in a change of data resolution. Minute-by-minute data from the SWA and AG were temporally matched and merged by participant ID, date and time. Processed data from the two monitors were linked to each participant’s demographic information (i.e. age, gender, and BMI). The SWA has the ability to precisely detect non-wear time periods since it collects data only when contacting the skin. In contrast, the AG relies on a series of prediction algorithms to identify periods of non-wear time. For the present study, the non-wear detecting algorithms by Choi et al. [23] were used. Matched minutes indicating non-wear from either monitor were dropped, and measured days with less than 10 h/day of valid data were removed from analysis [13]. Other common inclusion criteria (e.g. 3 valid days, 1 valid weekend day) were not applied, as they are tailored to surveillance studies and were thus not applicable to the present study.

After being processed in this manner, the data were further reduced by dropping days with less than 1000 or greater than 30,000 steps, in accordance with recommended procedures [24]. Screening resulted in a total loss of 72 measurement days distributed among 46 participants. The final sample included concurrent data obtained from the two monitors, with identical number of minutes and measurement days. Each participant provided at least one day of valid data, yielding a final sample of 69 children with a total of 476 measurement days. Detailed participant characteristics and descriptive statistics are provided in Table 1.

**Table 1:** Participant characteristics and descriptive statistics

	Total	Boys	Girls
N	69	44	25
Age (Years)	12.0 (2.2)	12.2 (2.3)	11.6 (2.0)
Height (cm)	155.9 (15.3)	158.6 (17.4)	151.1 (9.3)
Weight (kg)	47.0 (15.2)	49.3 (16.9)	43.1 (10.9)
Mean Daily Valid Minutes	824.1 (136.1)	819.2 (146.3)	832.7 (115.9)
BMI	18.9 (3.0)	19.0 (3.0)	18.6 (3.2)
Normal weight; n (%)	54 (78.3 %)	35 (50.7 %)	19 (27.5 %)
Overweight; n (%)	14 (20.3 %)	8 (11.6 %)	6 (8.7 %)
Obese; n (%)	1 (1.4 %)	1 (1.4 %)	0 (0.0 %)
MVPA minutes/day			
AG	120.7 (51.9)^a^	114.5 (51.0)^a^	131.6 (51.7)
SWA	155.4 (93.2)	161.4 (88.8)	144.7 (100.0)
Step counts/day			
AG	11,557 (4059)^b^	11,580 (4122)^b^	11,515 (3958)^b^
SWA	9930 (4456)	10,095 (4751)	9639 (3877)
Total measurement days	476	304	172

*Abbreviations AG* ActiGraph (Wrist-worn GT3X+), *BMI* Body Mass Index, *MVPA* Moderate-to-Vigorous Physical Activity (MVPA), *SWA* SenseWear Armband (Mini)

Note: Values in parentheses indicate standard deviations unless otherwise noted

^a^indicates significant differences between AG and SWA for MVPA minutes/day

^b^indicates significant differences between AG and SWA for Step counts/day

### Statistical analysis

Demographic factors were summarized, and means and standard deviations were calculated for the whole sample as well as demographic sub-groups. We first examined differences in MVPA minutes/day and step counts/day between the AG and SWA and between boys and girls, using multiple Two-Way mixed Analysis of Variance (ANOVA) tests to determine if differences were consistent across boys and girls. Main effects were tested for monitor and gender, with repeated measures for monitor. We were particularly interested in the boys vs. girls contrast in between-monitor comparisons. Statistical significance was set at an alpha of 0.05, but the alpha value was corrected in post-hoc analyses using Tukey’s Honestly Significant Difference method.

For the main analysis we defined valid measurement days as being the unit of observation. These analyses compared both 1) intra- and 2) inter-monitor classification agreement on the number of measurement days meeting PAG-MVPA and PAG-Steps. Analyses were computed using percent agreement and Cohen’s kappa (κ) statistics. This approach made it possible to examine 1) the impact of the selected outcome and 2) the impact of monitor choice (e.g. AG versus SWA), on the PAG prevalence rate regarding PAG-MVPA and PAG-Steps.

Percent agreement (a somewhat primitive measure) can be expressed as a proportion of total probability (i.e. observed agreement/1). Kappa statistics refine this approach by adjusting the numerator and denominator for the expected agreement due to chance. Thus, expected agreement is an informative parameter to include in agreement reports. A detailed explanation of its calculation is beyond the scope of this paper, but the reader is directed to Sim and Wright (2005) for more information [25]. Agreement was classified based on the following criteria proposed by Altman (1990) [26]: “Poor” (κ ≤ 0.20); “Fair” (0.21 ≤ κ ≤0.40); “Moderate” (0.41 ≤ κ ≤ 0.60); “Good” (0.61 ≤ κ ≤ 0.80); “Very Good” (0.8 ≤ κ ≤ 1.00). Statistical analyses were carried out jointly using R [27] and STATA 13 (StataCorp LP, College Station, TX).

## Results

A total of 476 measurement days from 44 boys and 25 girls had concurrent data on AG and SWA and were therefore deemed valid for analysis (Table 1). The first set of analyses demonstrated that the AG recorded fewer minutes of MVPA (F(1, 1) = 98.73, *p* < 0.0001), but more steps (F(1, 1) = 180.05, *p* < 0.0001), when compared to the SWA. These trends were consistent across boys and girls, and all were significant (*p* < 0.05) except minutes of MVPA for girls.

### Analysis set 1: agreement between activity outcomes

**Fig. 1:**
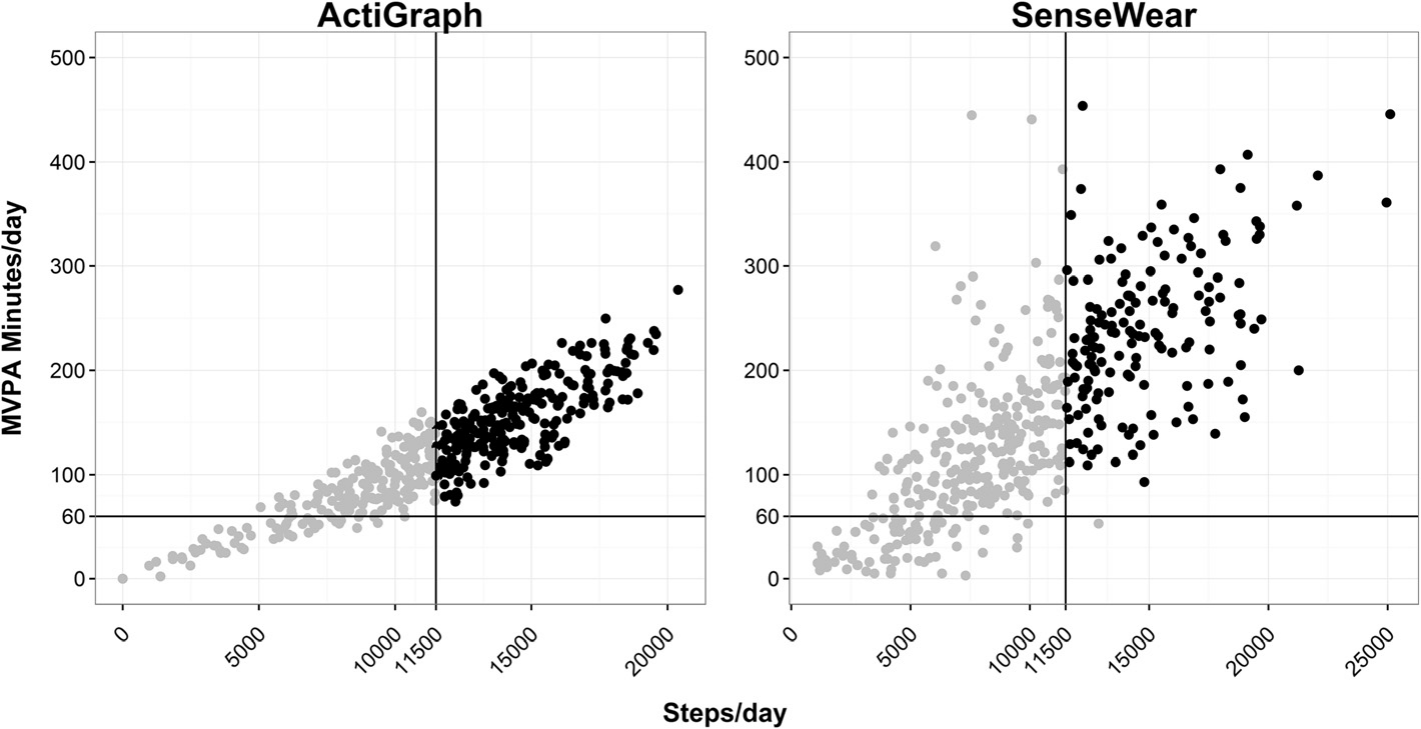
Compliance with guidelines for activity time versus steps for ActiGraph and SenseWear. Per-day moderate-to-vigorous physical activity (MVPA) is plotted on the y-axes, with per-day step counts on the x-axes, for ActiGraph (*left*) and SenseWear (*right*). Black dots indicate meeting both guidelines, while gray dots indicate meeting one or neither guideline

Figure 1 illustrates the differences in compliance between the guidelines for both instruments. Prevalence rates varied when PAG-MVPA or PAG-Steps were used. However, both monitors showed consistently higher prevalence of meeting the guidelines when PAG-MVPA was used. Compliance in meeting PAG differed by 33.0 % for the two indicators from the AG (PAG-MVPA = 87.2 % (415 days) versus PAG-Steps = 54.2 % (258 days)). The difference in compliance rates (49.8 %) was even larger with the SWA (PAG-MVPA = 83.6 % (398 days) versus PAG-Steps = 33.8 % (161 days)). The percent agreement between the measured number of days meeting the guidelines was 67 % for the AG and 49.8 % for the SWA and the classification agreement was fair for AG (κ = 0.30) and poor for SWA (κ = 0.18) (Table 2).

**Table 2:** Intra-monitor kappa statistics and percent agreement between the activity time guidelines with the step guidelines

	Kappa	Percent Agreement	Expected Agreement
AG	0.30	67.0 %	53.1 %
SWA	0.18	49.8 %	39.1 %

*Abbreviations AG* ActiGraph (Wrist-worn GT3X+), *SWA* SenseWear Armband (Mini)

### Analysis set 2: agreement between monitors

**Fig. 2:**
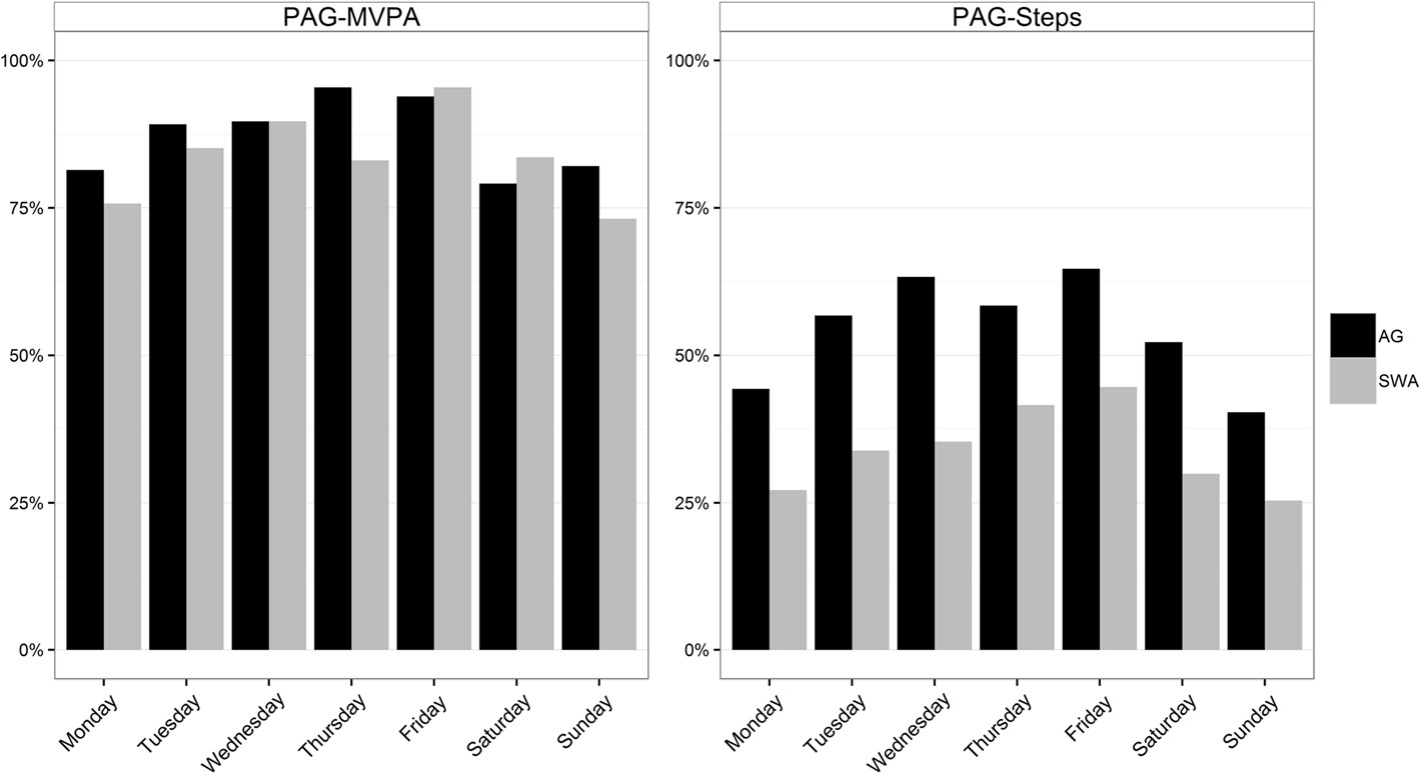
Summary of compliance with guidelines for activity time versus steps by day of the week. The trends throughout the week can be seen for the activity time guidelines (PAG-MVPA, left) and the step guidelines (PAG-Steps, right), as measured by the ActiGraph (AG) and the SenseWear Armband (SWA)

The two monitors differed by 3.6 % in their overall estimates of adherence to the PAG-MVPA, yet showed only moderate agreement in classifying individual days according to this guideline (Percent agreement = 85.5 %; κ = 0.42). The difference between the estimates of adherence to the PAG-Steps was slightly higher (4.4 %), and also exhibited moderate classification agreement (Percent agreement = 76.7 %; κ = 0.55) (Table 3). Figure 2 represents this visually by plotting prevalence across the days of the week, where the day-to-day relation of AG and SWA is more variable in PAG-MVPA measurements than PAG-Steps measurements.

**Table 3:** Inter-monitor kappa statistics and percent agreement for the activity time guidelines and the step guidelines

	Kappa	Percent Agreement	Expected Agreement
PAG-MVPA	0.42	85.5 %	75.0 %
PAG-Steps	0.55	76.7 %	48.6 %

*Abbreviations PAG-MVPA* activity time guidelines, *PAG-Steps* step guidelines

## Discussion

This study is the first empirical research to systematically investigate compliance in achieving PAG with different monitors and different metrics (i.e. PAG-MVPA and PAG-Steps). The primary findings were that: 1) agreement between the PAG-MVPA and the PAG-Steps was moderate when assessed by the AG and poor when assessed by the SWA, and 2) agreement between the AG and SWA in estimating adherence to the two guidelines was similar and moderate. These findings specifically suggest that surveillance of youth PA can be heavily influenced by both the choice of activity outcomes and the choice of measure. The results have implications not only for surveillance research, but also for further work in the validation and evaluation of guidelines assessed with objective monitors. For example, PAG have been proposed based on various criterion measures for both MVPA and steps (see, for instance, Table 2 of [14]). Our results underscore the potential implications of trying to assess compliance with different monitors and different measures, and the same limitations we found can be expected to exist in the use of these instruments as criteria for other applications.

The effort to establish step-based PAG that match existing MVPA-based guidelines is certainly important since steps are a popular indicator of activity and consumers have an easy time interpreting steps. Pedometers are inexpensive and accurate (many pedometers estimate steps with nearly perfect accuracy at most speeds [28]), so an equated relationship would make it possible to evaluate compliance with PAG more effectively [11]. However, the relationship between steps and MVPA has proven elusive despite various attempts to define it [11, 14, 19, 20, 29]. While the present study was primarily designed to examine the impact of instrumentation on these relationships, some qualitative observations are relevant. One interesting result is that, irrespective of the monitor used, only one case was found (measured by the SWA) in which the participant did not meet the PAG-MVPA and did meet the PAG-Steps (Fig. 1, lower right quadrant of SWA plot). Although not conclusive evidence, this consistent trend between both monitors seems to suggest that prevalence estimates from the PAG-Steps exhibit a high true positive rate.

It is not clear if the disparities between steps and MVPA are reflective of limitations related to instrumentation, or whether they are indicative of fundamental differences in the outcome measures. The former issue is more immediately evident. For example, the threshold proposed by Adams et al. [11] was established based exclusively on free-living uniaxial AG data, with corrections applied to step counts due to the heightened sensitivity of AG monitors at low levels of acceleration. In contrast, pedometers present an opposite challenge, as it is well established that they tend to record more steps for walking than running. While these issues point to methodological difficulties in establishing a set of definitive step-based thresholds, it should be noted that the main limitation may also be with the inherent nature of the step count indicator. For example, it is understood that pedometers tend to accrue more steps in individuals with smaller strides, hinting at the importance of differences between the outcomes. Therefore, it is possible that the unique nature of steps may prevent or limit the possible linkage between these measures, at least with the present approach.

A unique aspect of this study is that it was also possible to examine the relative agreement between two different monitors for a given metric. While many studies have examined measurement characteristics of activity monitors, the implications of choice of monitor and metric on evaluating compliance with PAG have not been examined.

The results demonstrate clear differences in outcomes that must be considered when interpreting data from objective monitoring devices. A contributing factor to this problem is the lack of consensus about the most effective methods for standardizing output and processing data. Several cut points are available for assessing activity intensity using wrist-worn AGs in youth [21]. One method is based on placement at the dominant wrist and the other on the non-dominant wrist; however it is unclear whether this distinction is even necessary [10]. Our evaluation of the two methods has revealed only minor differences in estimates from these two methods regardless of handedness (unpublished observations). Justification for using the Crouter method was provided earlier but additional research is needed to determine the specific differences between these tools and the implications of wearing monitors on the dominant or non-dominant wrist.

The lack of clarity regarding the best method for processing wrist-worn AG data is only one of many questions that must be resolved regarding the use of AG monitors. Progress towards refining an ideal method was set back considerably when AG monitors transitioned from being worn at the hip to being worn at the wrist. Additional challenges have also emerged with the development of methods for processing the newly-available raw acceleration data [30]. The changes may ultimately be beneficial but systematic evaluations are needed to evaluate differences between monitors and between methods.

Research regarding the validity of step count estimates from the AG and SWA is even more limited, particularly for children and adolescents. The wrist attachment used for the AG is an issue of equal (if not greater) implication for step counts as MVPA. Such placement has been an area of some debate for several reasons, which include the possibility that wrist-worn monitors produce artefactual step recordings during non-ambulatory daily activities such as eating or writing [20], as well as the possibility that the acceleration profiles reflective of stepping at the waist versus the hip are too dissimilar to be governed by a single step-detecting algorithm [31]. The latter concern may be supported by the findings of a recent study, which showed that wrist-worn AG monitors filtered with the default filter recorded higher daily step counts (by an average of 2558 steps/day) in free-living adults than waist-worn monitors [21]. Currently, no such evidence currently exists for youth populations.

The step count function of the SWA has received comparatively little attention in healthy and/or youth populations, with accentuated gaps in research on more recent generations. For example, Arvidsson et al. [17] found consistent underestimation of steps in healthy African American children, 11 of normal BMI and 15 of high BMI. However, the underestimation of SWA step count was only significant at a walking speed of 2 km/h in the normal BMI children. Equivocal trends have been seen in existing studies for adults, but underestimation has been frequently observed in these samples as well [32, 33]. A systematic review by Van Remoortel et al. [34] reported that slow walking produced low step count estimates from all accelerometers, with some improvement at higher speeds. The universal applicability of this trend is unclear, however. Most notably, Lee and Laurson [35] recently published the first SWA step count validity study in healthy adults wearing a precursor to the model used in the present study. Interestingly, their findings paralleled the findings of Tudor-Locke et al. [28] for wrist-worn AG monitors, in that step counts in the laboratory were underestimated by 5.6–16.0 % at various walking speeds, compared to a criterion (manual step count), but were overestimated by 11.3 % in free-living, relative to an established comparison measure (Yamax Digiwalker pedometer). Additional research is clearly needed to investigate the step function of both the wrist-worn AG and SWA relative to a strong criterion measure for diverse youth populations. To our knowledge, no such criterion validity study has been carried out for both monitors.

This study provides some unique comparisons but there are limitations that should be noted when interpreting the findings. First, there was no criterion measurement method used herein, so it was not possible to draw conclusions about the relative accuracy of the different monitors. The placement of the AG at the wrist could also be viewed as a limitation since the threshold for PAG-Steps was based on data at the hip. However, these limitations did not affect our primary objectives with the paper. We sought to compare methods and measures in order to advance understanding of how these factors can influence outcomes in other studies. Choices about attachment site are a highly relevant issue in accelerometer research, and the wrist attachment allowed a more contemporary (and thereby impactful) illustration of the consequences of moving to this location for surveillance applications.

## Conclusions

The results of the present study underscore the impact of outcome and instrument choice on both the development of guidelines for sufficient PA in youth, and the conclusions drawn from surveillance research that implements these guidelines. Overall, the two widely-used activity tracking devices exhibited relatively poor agreement in classifying individuals as meeting the PAG-MVPA or PAG-Steps. This considerable discrepancy suggests that public health researchers should pay particular attention to designing, implementing and/or evaluating epidemiological surveillance research to correctly determine the prevalence of meeting the PAGs in children and adolescents. Additional research is required to examine the utility and accuracy of activity monitors for assessing activity time and step counts, and its implications on evaluating adherence to published activity guidelines in youth populations.
